# Network Meddling Detection Using Machine Learning Empowered with Blockchain Technology

**DOI:** 10.3390/s22186755

**Published:** 2022-09-07

**Authors:** Muhammad Umar Nasir, Safiullah Khan, Shahid Mehmood, Muhammad Adnan Khan, Muhammad Zubair, Seong Oun Hwang

**Affiliations:** 1 Riphah School of Computing & Innovation, Faculty of Computing, Riphah International University, Lahore Campus, Lahore 54000, Pakistan; 2 Department of IT Convergence Engineering, Gachon University, Seongnam 13120, Korea; 3 Pattern Recognition and Machine Learning Lab, Department of Software, Gachon University, Seongnam 13557, Korea; 4 Faculty of Computing, Riphah International University, Islamabad Campus, Islamabad 45000, Pakistan; 5 Department of Computer Engineering, Gachon University, Seongnam 13120, Korea

**Keywords:** machine learning, network security, meddling detection, cyber attack

## Abstract

The study presents a framework to analyze and detect meddling in real-time network data and identify numerous meddling patterns that may be harmful to various communication means, academic institutes, and other industries. The major challenge was to develop a non-faulty framework to detect meddling (to overcome the traditional ways). With the development of machine learning technology, detecting and stopping the meddling process in the early stages is much easier. In this study, the proposed framework uses numerous data collection and processing techniques and machine learning techniques to train the meddling data and detect anomalies. The proposed framework uses support vector machine (SVM) and K-nearest neighbor (KNN) machine learning algorithms to detect the meddling in a network entangled with blockchain technology to ensure the privacy and protection of models as well as communication data. SVM achieves the highest training detection accuracy (DA) and misclassification rate (MCR) of 99.59% and 0.41%, respectively, and SVM achieves the highest-testing DA and MCR of 99.05% and 0.95%, respectively. The presented framework portrays the best meddling detection results, which are very helpful for various communication and transaction processes.

## 1. Introduction

A network invasion is a brief interruption in regular network activity. Some outbreaks are maliciously produced by attackers, such as a denial of service (DoS) assault on an internet protocol (IP) network, while others are entirely accidental, such as an overpass collapsing into a busy road network [1]. Rapid detection is required to launch a rapid reaction, such as dispatching an ambulance following a traffic accident or sounding an alert when a surveillance network detects an intruder. Data are collected at a rapid pace by network monitoring equipment. As a result, developing an efficient anomaly detection system entails extracting important information from enormous amounts of noisy and big data. It is also critical to develop distributed algorithms since networks have bandwidth and power restrictions, and communication costs must be kept to a minimum. With the sophistication and innovation of technology, software, and network topologies, cyber intrusions continue to evolve. To protect the network from hostile cybersecurity threats, intrusion detection systems are highly recommended [1]. There are firewalls and other rule-based security mechanisms in place, which are commonly utilized to safeguard contemporary data centers against network threats. Large distributed multi-cloud systems, on the other hand, would need a large number of complicated rules, which might be costly, time-consuming, and error-prone [2]. Furthermore, developments in computing have enabled attackers to scale attacks, such as the emergence of distributed DoS (DDoS) attacks, which are seldom detected by ordinary firewalls [3]. As a result, using a firewall alone is insufficient to offer total system security in multi-cloud environments.

Deep neural networks can learn complicated patterns of abnormalities directly from network traffic data; therefore, machine learning approaches have attracted a lot of attention in recent years [4]. Real-world traffic data, on the other hand, are large-scale, noise-labeled, and imbalanced in class. In other words, there are millions of samples in the traffic data that are unevenly distributed, with infrequent abnormalities and far too much typical traffic data. The majority of current network datasets do not fulfill real-world requirements and are therefore unsuitable for modern networks. Furthermore, conventional datasets, such as kddcup99 [5] and UNSW-NB15 [6], have received a lot of attention in the literature. These datasets have enabled methods to achieve great throughput. As a result, in this study, we focus on the problem of large-scale (millions of scales) and highly imbalanced traffic data by training, validating, and testing the suggested solution using the ZYELL dataset [7,8].

In this paper, the data were collected using IoT technology, which can access data from different cloud environments including vehicular networks and numerous cooperation. Thus, the collected data were in massive amounts and transmitted using IoT technology without any delays and lag toward the data pre-processing layer and were then saved in blockchain technology-secured clouds.

The novel proposed model in this study uses machine learning algorithms. These machine learning algorithms are utilized to detect meddling and normal communication transactions. The proposed model uses blockchain technology to overcome the security threats of trained models and data preprocessing. Thus, the proposed model achieved the highest detection results with the help of machine learning and blockchain technology.

## 2. Literature Review

A great number of research studies on IDS employing machine learning approaches have been published in the literature. For example, the authors of [9,10] employed support vector machines (SVMs) to find abnormalities in the KDD dataset [5]. The authors of [11] constructed IDS models for anomaly detection using artificial neural networks utilizing the same dataset. The author of [12] employed cascade classifiers to detect and classify anomalies in the KDD dataset, even when they were spread unevenly. References [13,14] suggested utilizing decision trees and random forest (RF) to discover anomalies. Furthermore, the authors of [15] presented hybrid approaches that make use of two or more machine learning algorithms. In terms of performance, the results suggest that hybrid strategies outperform individual models. For further information on machine learning methodologies in IDS, readers can refer to the surveys provided in [16,17,18].

The SVM is used for early classification and regression problems [19]. The SVM belongs to the linear generalized classification family and has the special property of simultaneously increasing the geometric margin and decreasing the empirical classification error, so in this manner, the SVM is also known as the maximum margin classifier. The SVM inputs its features or vector in the maximum higher separated hyperplane with a higher dimensional space. For the separation of data, parallelly, two hyperplanes were constructed. These hyperplanes could increase the distance between these binary hyperplanes. The SVM considers the data points {(x_1_,y_1_), (x_2_,y_2_), (x_3_,y_3_), (x_4_,y_4_)… (x_n_,y_n_)} where each x_n_ is considered as the input vector.

KNN is a scenario-learning technique [20] that makes classification choices using all of the training data. It is unsuitable for many applications due to its sluggish learning rate, such as dynamic web mining for a large collection. Finding some representatives to represent all of the training data for classification is a strategy to improve its efficiency; that is, create an inductive learning model from the training dataset and utilize this model (representatives) for classification. Many modern algorithms, such as DT and NN, were designed to produce such a model. One assessment factor involves the efficiency of various algorithms. Thus, we chose these two machine learning algorithms because the SVM is a parametric linear solver algorithm, and most of its success depends on tuning parameters that decrease the overall generalization error. KNN is cost-effective (for classification) and can single-handedly handle large non-parametric datasets.

The authors of [21] employed a four-layer classification algorithm on the KDD dataset to detect four types of assaults. The findings indicate a modest classification mistake and a minor overall inaccuracy. In addition, the scientists claimed that the number of features in the original dataset was reduced, resulting in lower complexity and higher accuracy. However, the authors found no misclassification as a result of misclassifying one type of violence as another. Using the same dataset and typical unsupervised and supervised learning techniques, such details were described in [22]. Several attacks were misclassified, resulting in poorer overall accuracy than the research presented in [21].

The KDD dataset has been extensively utilized to train machine learning models for anomaly detection and classification. The KDD dataset contains four different types of assaults with radically different traffic characteristics. Reference [21] presents a technique for the categorization of assaults using KDD that achieved remarkably low misclassification errors. However, such models may not be suitable for today’s numerous cloud situations with varying forms of evolution and closely related assaults. Furthermore, the KDD dataset is aging and may no longer reflect current real-time traffic patterns and network threats [23]. Furthermore, if a new assault is launched, it will most likely go unreported, resulting in a high mistake rate, as documented in [22].

Machine learning has been used to improve network security in a variety of settings. Buczak et al. [24] and Hodo et al. [25] focused on malware detection in the cybernetwork using supervised and unsupervised learning. DaCosta et al. [26] examined intrusion detection in the context of IoT utilizing machine learning applications. Ucci et al. [27] and Tahsien et al. [28] and Hussain et al. [29] addressed the possible dangers of the IoT and proposed machine learning methods to address these issues. Gibert et al. [30] used supervised and unsupervised learning to identify and classify malware in a Windows system. Nassif et al. [31] investigated cloud network dangers and how to defend the cloud network using supervised learning.

Machine learning may be used in network administration to avoid possible attacks in addition to detecting anomalous activity. Jin et al. [32] used reinforcement learning to determine the appropriate scheduling method for managing intranet traffic while keeping security in mind. Every user has a reputation value that indicates how reliable their traffic is. The available bandwidth of the connections and the flows that are waiting to be scheduled describe the reinforcement learning condition. In the suggested approach, actions are assigned to each stream, and each action is made up of the bandwidth allocated to that stream. Link utilization, queue length, latency, and the user’s trust level all contribute to the scheduler’s performance.

Table 1 shows the previous studies’ limitations and methodologies. Jin et al. [32] used reinforcement learning to detect insider threats using a public dataset; it achieved 98% accuracy but data preprocessing and imbalanced classes were its limitations. Hamamato et al. [33] used k-means to detect DDoS and DoS attacks using a public dataset; it achieved 96.53% accuracy but less performance in a supervised manner and with imbalanced classes. Gu et al. [34] used k-means to detect DDoS attacks using a public dataset; it achieved 98.9% accuracy but a high recall detection and lack in classification. Alauthman et al. [35] used a neural network to detect botnet attacks using a public dataset; it achieved 98.3% accuracy but data preprocessing and imbalanced classes were its limitations. Smadi et al. [36] used a neural network to detect phishing threats using a public dataset; it achieved 98.6% accuracy but machine learning and imbalanced classes were its limitations. Xu et al. [37] used Q-learning to detect general network threats using a public dataset and achieved 95% accuracy; however, data preprocessing and imbalanced classes were its limitations. Sethi et al. [38] used reinforcement learning to detect DoS threats using a public dataset and achieved 97.8% accuracy; however, preprocessing features and imbalanced classes were its limitations. Rashid et al. [39] used numerous machine learning approaches, including SVM, DT, KNN, RF, LR, etc., to detect cyber-attacks in IoT-based smart city applications. They used UNSW-NB15 and CICID2017 datasets to detect cyber-attacks. Their system achieved more than 95% cyber-attack detection accuracy but data fuzzing and feature engineering were its limitations. Ofori et al. [40] used machine learning to predict threats at early stages with the help of LR, DT, NB, and RF, achieving 70% performance in predicting cyber threats; however, data control and attributions were the limitations.

The major contributions of this study are as follows:

- The proposed framework employed machine learning approaches to detect network attacks.
- The proposed model used blockchain technology to secure the trained models and communicated data.
- The proposed study used numerous statistical parameters to evaluate the performance and authenticity of models.

## 3. Materials and Methods

### 3.1. Blockchain Module

A blockchain block comprises a massive amount of transaction information, including the block ID, a hash of the previous block, transaction information, null byte, and headers. In a blockchain system where prospectors select the appropriate hash to add a block to, contenders check for a current block before looking for a new one. The proof-of-work process is used to determine the legitimacy of a certain block of transactions. The phases that follow illustrate the essential parts of blockchain technology. Any node in a computerized health system that is connected to the internet must communicate with a storage database as well as settlers on a private blockchain. Unprocessed transactions are stored in the blockchain until a new block is assigned for authentication. Many transactions are examined before being quickly processed by the Merkel tree, a binary hash tree. Blockchain technology will be utilized to develop a new smart medical system connection biosphere since it is diversified and compatible with the internet of medical things applications. The blockchain module provides the proposed model with the following protection.

**Onset:** To begin implementing data protection parameters, the cryptographic algorithms must first be concocted.

**Encrypting:** The user defines the *block index* and the *proceeding detail*, where the *block index* is defined as the *box index* and the *proceeding detail* as plaintext. Thus, it gives the proposed model *Encrypt *(*index*, *text*)** value.

**Encampment:** The proposed model contains a *box index* unit, after which the machine learning model delivers security keys $Security\_Block-Z_{index}$.

**Defy:** It only allows one to complete the full iteration, initiate (create), and send two messages (text1, text2) for each index ∈ Z. After completing the iteration and calculation, if it has 0, then $Security\_Block_{p}=Encrypt\left(memory\right)$, or else it repeats the full iteration.

### 3.2. Anomaly Detection Module

**Figure 1:**
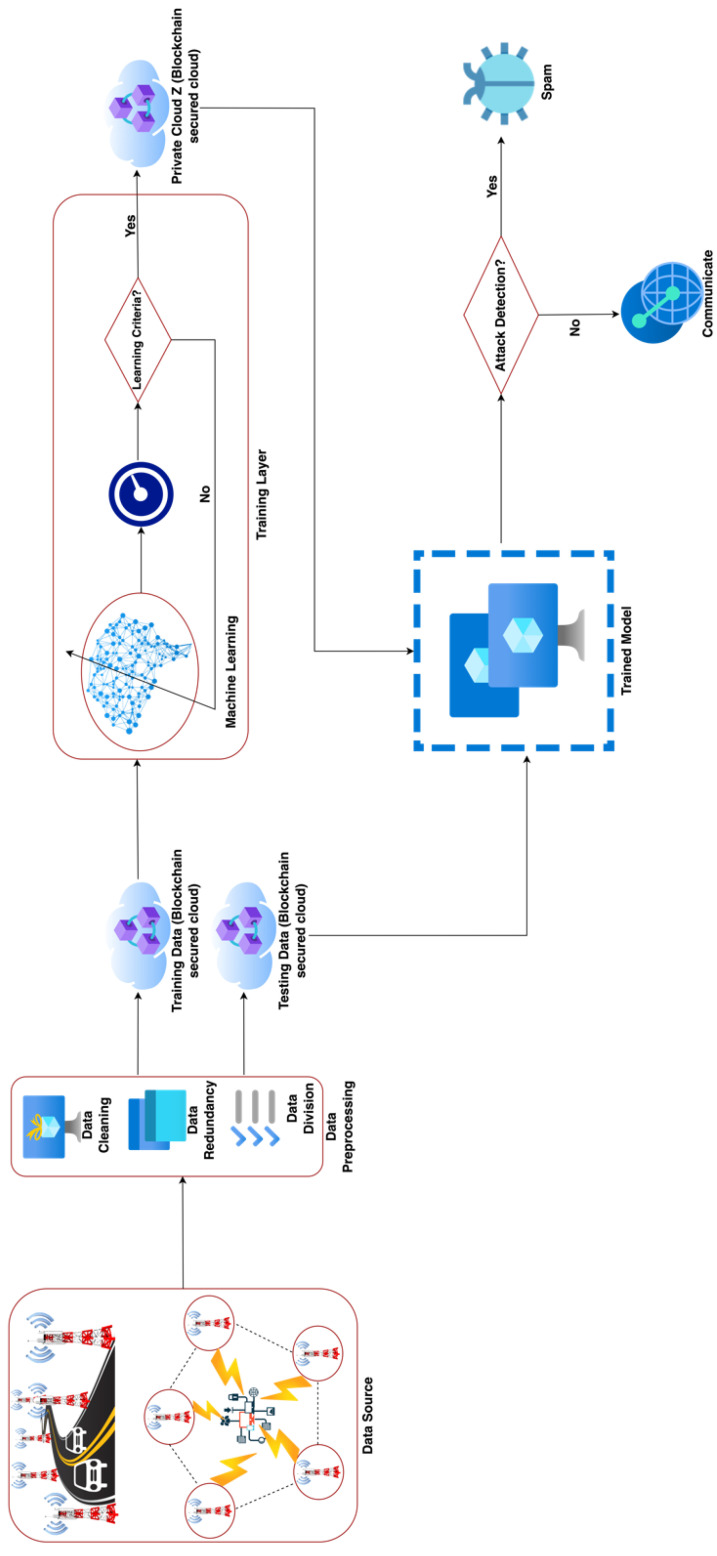
Proposed model for network meddling detection using machine learning entangled with blockchain technology.

Figure 1 depicts the overall picture of general network meddling detection, empowered with machine learning, and entangled with blockchain technology. The proposed model consists of four phases. At the initial stage, the proposed model collects data using the Internet of Things (IoT) from the vehicular network and general network communication transactions using PyShark and passes the raw data toward the data preprocessing phase. In this phase, the proposed model applies data cleaning techniques, including unwanted outliers, fixing data structural errors with swift observations to save the time–costs of the model and data redundant techniques, cleaning the data from a bug or null values, and fixing duplicate and missing values. Right after the data pre-processing, the proposed model applies the feature extraction technique with the help of embedded methods and then stores preprocessed data into blockchain-entangled private clouds for further processing. The third stage of the proposed model-training layer imports data from a private cloud of training data to train machine learning algorithms (SVM and KNN) and check the trained model performance. If the learning criteria meet, then the model is stored in a private cloud, *Z*, otherwise it retrains the process. In the final phase, the testing layer imports the trained model from the private cloud, *Z*, and imports the testing data from the private cloud; then the testing process starts. If the network meddling detects, then the data are processed into spam, otherwise, the data are processed for further communications and transactions. To evaluate the overall performance of testing, the proposed model applies various statistical parameters [40,41,42,43,44,45,46,47,48,49,50], i.e., detection accuracy (DA), sensitivity, F1-score, specificity, likelihood negative ratio (LNR), false positive rate (FPR), false negative rate (FNR), misclassification rate (MCR), likelihood positive ratio (LPR), positive predicted value (PPV), and negative predicted value (NPV) to check the performance of the proposed framework. All equations are explained below.

(1) $$\mathrm{DA}=\frac{á\;+\;ß}{á\;+\;ß+ð+Ø}\;\times \;100$$

(2) $$\mathrm{MCR}=100-\frac{á\;+\;ß}{á\;+\;ß+ð+Ø}\times 100$$

(3) $$\mathrm{Sensitivity}=\;\frac{á\;}{á\;+Ø}\times 100$$

(4) $$\mathrm{Specificity}=\;\frac{ß}{ß+ð}\times 100$$

(5) $$F1-\mathrm{score}=\frac{2á\;}{2á\;+\;ð+Ø}\times 100$$

(6) $$\mathrm{PPV}=\;\frac{á\;}{á\;+\;ð}\times 100$$

(7) $$\mathrm{NPV}=\;\frac{\;ß}{ß+Ø}\times 100$$

(8) $$\mathrm{FPR}=100-\;\frac{ß}{ß+ð}\times 100$$

(9) $$\mathrm{FNR}=100-\;\frac{á\;}{á\;+Ø}\;\times \;100$$

(10) $$\mathrm{LPR}=\frac{\frac{á\;}{á\;+\;Ø}\times 100}{100-\;\frac{ß}{ß\;+\;ð}\times 100}$$

(11) $$\mathrm{LNR}=\frac{100-\;\frac{á\;}{á\;+\;Ø}\times 100}{\frac{ß}{ß\;+\;ð\;}\times 100}$$

The computational complexities of the two classification algorithms utilized in this work are briefly discussed below:

The KNN algorithm does not memorize training data; rather, it performs the nearest neighbor calculation online. The algorithm calculates the distance between each test and training sample. Once all distances are computed, they are arranged in ascending order to identify the nearest neighbor. Each training sample is a spot in the attribute space if there are m attributes in the training data, making the attribute space m-dimensional. The computational complexity of the KNN algorithm depends on the number of features (*m*), the number of training samples (*n*), and an odd number chosen as the value of ‘k’. The following Euclidian distance formula is most frequently used to calculate distances.

(12) $$D_{ij^{2}}\overset{m}{\underset{v=1}{\sum }}{\left(X_{vi}-X_{vj}\right)}^{2\;}$$

Since *m* multiplications are required to calculate the distance between each training sample and the test sample, the KNN Euclidean distance-based complexity is O (*mn*). Complicating factors include distance sorting. The merge–sort algorithm, for instance, uses O (*n* log *n*). Therefore, the overall complexity is O (*mn* + *n* log *n*). In an m-dimensional feature space, if ***A*** and ***B*** are two points in the feature space and constitute feature vectors ***A*** = (y1, y2, …, ym) and ***B*** = (z1, z2, …, zm), respectively, the cosine distance between them is given by

(13) $$sim\left(A,\;B\right)=\frac{\vec{A\;}\cdot \vec{\;B}}{\vec{\left|A\right|}\;\vec{\left|B\right|}}$$

It will take m multiplications to compute the dot product in the numerator. Each vector will need to be multiplied m times in order to determine its Euclidian length in the denominator. Therefore, to calculate the similarity, 3 *m* multiplications are required. The total complexity is O (3*mn* + *n* log *n*) when the sorting algorithm’s complexity is taken into account [51].

The SVM is frequently used to effectively address both regression and classification problems. The SVM accuracy is generally greater than KNN accuracy. However, both the complexity and training time of the SVM are greater than those of KNN. Abdiansah et al. display the complexity of SVM as O (*n*^3^) in [52].

## 4. Dataset

The proposed model used the online UNR-IDD [53] available network meddling data for the training and testing phases due to its relevance to cyber-attacks and anomaly detection; moreover, it is more relevant to network communication. This dataset is most suitable for IoT. The dataset consists of two classes—attack and normal—and has 37,000 instances with 30 dependent and 1 independent variable. Table 2 shows the description of the selective variables of the dataset.

## 5. Simulation and Results

In this research, the proposed framework employed ML for the detection of network meddling empowered with blockchain technology. For the simulation, including training and testing the data, the proposed model used MacBook Pro 2017, 512 GB SSD, 16 GB, Core i5, and embedded GPU. To remove the major discrepancies in data, the proposed model applied numerous data preprocessing techniques and stored preprocessed data in private clouds. The proposed framework employed SVM and KNN machine learning algorithms to train and test network meddling data. All phases of the proposed model were discussed in this research. The proposed framework applied various statistical performance parameters to measure the performance and authenticity of the model.

**Figure 2:**
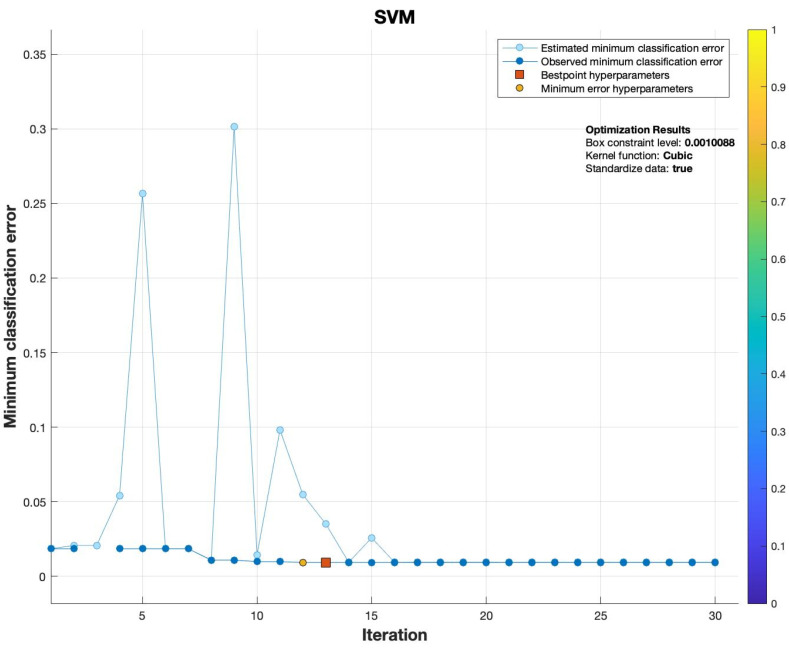
Training MCE of SVM for the detection of network meddling.

Figure 2 shows the training performance of the SVM. It depicts the minimum classification error (MCE) of SVM at 30 iterations; optimized results of the SVM show a box constraint level of 0.0010088 with a kernel function cubic, achieving MCE 0.0091778 at the 13th iteration (which was best among all models).

Table 3 shows the training confusion matrix of the SVM. The proposed model detected 27,190 attack instances and 2290 normal instances; it wrongly detect 120 instances.

Table 4 shows the statistical parameter results of training the SVM; the proposed model achieved 99.59%, 0.41%, 99.96%, 95.42%, 99.78%, 99.60%, 99.57%, 4.58%, 0.04%, 21.81%, 0.00, and 99.78% of DA, MCR, Sen, Spec, F1-score, PPV, NPV, FPR, FNR, LPR, LNR, and FMI, respectively.

Table 5 shows the training confusion matrix of KNN. The proposed model detected 27,190 attack instances and 2200 normal instances; it wrongly detected 220 instances.

Table 6 shows the statistical parameter results of training KNN; the proposed model achieved 99.29%, 0.71%, 99.63%, 95.24%, 99.62%, 99.60%, 99.65%, 4.76%, 0.37%, 20.92%, 0.00, and 99.62% of DA, MCR, Sen, Spec, F1-score, PPV, NPV, FPR, FNR, LPR, LNR, and FMI, respectively.

Table 7 shows the testing confusion matrix of SVM. The proposed framework detected 6400 attack instances and 930 normal instances; the proposed model wrongly detected 70 instances.

Table 8 shows the statistical parameter results of testing the SVM; the proposed model achieved 99.05%, 0.95%, 99.84%, 93.94%, 99.46%, 99.07%, 98.94%, 6.06%, 0.16%, 16.47%, 0.00, and 99.46% of DA, MCR, Sen, Spec, F1-score, PPV, NPV, FPR, FNR, LPR, LNR, and FMI, respectively.

Table 9 shows the testing confusion matrix of KNN. The proposed model detected 6400 attack instances and 850 normal instances; it wrongly detected 150 instances.

Table 10 shows the statistical parameter results of testing KNN; the proposed model achieved 97.97%, 2.03%, 99.84%, 85.86%, 98.84%, 97.86%, 98.84%, 14.14%, 0.16%, 7.06%, 0.00, and 98.85% of DA, MCR, Sen, Spec, F1-score, PPV, NPV, FPR, FNR, LPR, LNR, and FMI, respectively.

Table 11 depicts the comparative studies of the current research with previous studies. Jin et al. [32] used reinforcement learning to detect insider threats using a public dataset, achieving 98% accuracy. Hamamato et al. [33] used k-means to detect DDoS and DoS attacks using a public dataset, achieving 96.53% accuracy. Gu et al. [34] used k-means to detect DDoS attacks using a public dataset, achieving 98.9% accuracy. Alauthman et al. [35] used a neural network to detect botnet attacks (using a public dataset), achieving 98.3% accuracy. Smadi et al. [36] used a neural network to detect phishing threats (using a public dataset), achieving 98.6% accuracy. Xu et al. [37] used Q-learning to detect general network threats (using a public dataset), achieving 95% accuracy. Sethi et al. [38] used reinforcement learning to detect DoS threats (using a public dataset), achieving 97.8% accuracy. Rashid et al. [39] used numerous machine learning approaches (including SVM, DT, KNN, RF, LR, etc.) to detect cyber-attacks in IoT-based smart city applications. They used UNSW-NB15 and CICID2017 datasets to detect cyber-attacks. Their system achieved more than 95% cyber-attack detection accuracy. Ofori et al. [40] used machine learning to predict threats at early stages with the help of LR, DT, NB, and RF. They achieved 70% accuracy in predicting cyber threats; the proposed model used machine learning algorithms to detect general network meddling, achieving 99.05% DA, which was the highest when compared to others. The proposed model also provided a blockchain-secured cloud for models and communication data.

## 6. Conclusions and Future Work

Machine learning has a primary role in the detection of meddling in vehicular and general network communications and transactions. Machine learning can help to prevent meddling problems at the run time and before they occur. In this research, the proposed framework used various machine learning techniques; the SVM outperformed all, achieving 99.05% DA and 0.95% MCR (empowered with blockchain technology) to overcome the security threats to trained models and network communication and transaction data. In the future, we intend to apply federated machine learning empowered with fuzzy data to overcome more problems regarding network meddling.

## Figures and Tables

**Table 1:** Limitation of previous studies for meddling detection in the network.

Paper	Model	Dataset	Anomaly	Blockchain	Detection Accuracy (%)	Limitation
Jin et al. [32]	Reinforcement learning	Public	Insider threats	No	98%	Preprocessing, imbalanced classes
Hamamoto et al. [33]	K-means	Public	DDoS, DoS	No	96.53%	Less performance in a supervised manner, imbalanced classes
Gu et al. [34]	K-means	Public	DDoS	No	98.9%	High in recall detection but lacks classification
Alauthman et al. [35]	Neural Network	Public	Botnet	No	98.3%	Preprocessing, imbalanced classes
Smadi et al. [36]	Neural Network	Public	Phishing	No	98.6%	Machine learning processing, imbalanced classes
Xu et al. [37]	Q-Learning	Public	General network	No	95%	Preprocessing, imbalanced classes
Sethi et al. [38]	Reinforcement Learning	Public	DoS	No	97.8%	Preprocessed features, imbalanced classes
Rashid et al. [39]	Machine learning	Public	Cyber attack	No	95%	Data fuzzing and feature engineering
Ofori et al. [40]	Machine learning	Public	Cyber threat	No	70%	Data control and attributions

**Table 2:** Description of the dataset.

Variable	Type	Variable	Type
Received packet	Categorical	Received bytes	Categorical
Sent packets	Categorical	Sent bytes	Categorical
Port alive duration	Categorical	Packets RX dropped	Categorical
Packets TX dropped	Categorical	Packets RX errors	Categorical
Packets Tx errors	Categorical	Delta received packets	Categorical
Delta received bytes	Categorical	Delta sent bytes	Categorical

**Table 3:** Training confusion matrix of SVM.

Total Instances (29,600)	Attack	Normal
**Attack**	27,190	110
**Normal**	10	2290

**Table 4:** Performance Analysis of SVM (%) During Training Phase.

DA	MCR	Sen	Spec
99.59	0.41	99.96	95.42
**F1**	**PPV**	**NPV**	**FPR**
99.78	99.60	99.57	4.58
**FNR**	**LPR**	**LNR**	**FMI**
0.04	21.81	0.00	99.78

**Table 5:** Training confusion matrix of KNN.

Total Instances (29,600)	Attack	Normal
**Attack**	27,190	110
**Normal**	100	2200

**Table 6:** Performance Analysis of KNN (%) During Training Phase.

DA	MCR	Sen	Spec
99.29	0.71	99.63	95.24
**F1**	**PPV**	**NPV**	**FPR**
99.62	99.60	95.65	4.76
**FNR**	**LPR**	**LNR**	**FMI**
0.37	20.92	0.00	99.62

**Table 7:** Testing confusion matrix of the SVM.

Total Instances (7400)	Attack	Normal
**Attack**	6400	60
**Normal**	10	930

**Table 8:** Performance Analysis of SVM (%) During Testing Phase.

DA	MCR	Sen	Spec
99.05	0.95	99.84	93.94
**F1**	**PPV**	**NPV**	**FPR**
99.64	99.07	98.94	6.06
**FNR**	**LPR**	**LNR**	**FMI**
0.16	16.47	0.00	99.46

**Table 9:** Testing confusion matrix of KNN.

Total Instances (7400)	Attack	Normal
**Attack**	6400	140
**Normal**	10	850

**Table 10:** Performance Analysis of KNN (%) During Testing Phase.

DA	MCR	Sen	Spec
97.97	2.03	99.84	85.86
**F1**	**PPV**	**NPV**	**FPR**
98.84	97.86	98.84	14.14
**FNR**	**LPR**	**LNR**	**FMI**
0.16	7.06	0.00	98.85

**Table 11:** Comparative analysis with previous studies.

Paper	Model	Dataset	Anomaly	Blockchain	Detection Accuracy (%)
Jin et al. [32]	Reinforcement learning	Public	Insider threats	No	98%
Hamamoto et al. [33]	K-means	Public	DDoS, DoS	No	96.53%
Gu et al. [34]	K-means	Public	DDoS	No	98.9%
Alauthman et al. [35]	Neural Network	Public	Botnet	No	98.3%
Smadi et al. [36]	Neural Network	Public	Phishing	No	98.6%
Xu et al. [37]	Q-Learning	Public	General Network	No	95%
Sethi et al. [38]	Reinforcement Learning	Public	DoS	No	97.8%
Rashid et al. [39]	Machine learning	Public	Cyber-attack	No	95%
Ofori et al. [40]	Machine learning	Public	Cyber threat	No	70%
**The Proposed Model**	**Machine Learning**	**Public**	**General Network**	**Yes**	**99.05%**

## Data Availability

The simulation files/data used to support the findings of this study are available from the corresponding author upon request.

## References

[1] Vinayakumar R., Alazab M., Soman K.P., Poornachandran P., Al-Nemrat A., Venkatraman S. (2019). Deep Learning Approach for Intelligent Intrusion Detection System. IEEE Access.

[2] Hu H., Ahn G.J., Kulkarni K. (2012). Detecting and Resolving Firewall Policy Anomalies. IEEE Trans. Dependable Secur. Comput..

[3] Hayajneh T., Mohd B.J., Itradat A., Quttoum A.N. (2013). Performance and information security evaluation with firewalls. Int. J. Secur. Its Appl..

[4] Maya S., Ueno K., Nishikawa T. (2019). dLSTM: A new approach for anomaly detection using deep learning with delayed prediction. Int. J. Data Sci. Anal..

[5] KDD Cup 1999 Data. http://kdd.ics.uci.edu/databases/kddcup99/kddcup99.html.

[6] The UNSW-NB15 Dataset. https://research.unsw.edu.au/projects/unsw-nb15-dataset.

[7] https://nad2021.nctu.edu.tw/Dataset.html.

[8] Chen L., Weng S.-E., Peng C.-J., Shuai H.-H., Cheng W.-H. Zyell-Nctu Nettraffic1.0: A Large-Scale Dataset for Real-World Network Anomaly Detection. https://arxiv.org/abs/2103.05767.

[9] Kim D.S., Nguyen H.N., Park J.S. Genetic algorithm to improve SVM based network intrusion detection system. Proceedings of the 19th International Conference on Advanced Information Networking and Applications (AINA’05) Volume 1 (AINA Papers).

[10] Guang Y., Min N. Anomaly intrusion detection based on wavelet kernel LS-SVM. Proceedings of the 2013 3rd International Conference on Computer Science and Network Technology.

[11] Kumar S., Yadav A. Increasing performance of intrusion detection system using neural network. Proceedings of the 2014 IEEE International Conference on Advanced Communications, Control and Computing Technologies.

[12] Khor K.C., Ting C.Y., Phon-Amnuaisuk S. (2012). A cascaded classifier approach for improving detection rates on rare attack categories in network intrusion detection. Appl. Intell..

[13] Stein G., Chen B., Wu A.S., Hua K.A. (2005). Decision tree classifier for network intrusion detection with GA-based feature selection. Proceedings of the 43rd Annual Southeast Regional Conference-Volume 2.

[14] Tesfahun A., Bhaskari D.L. Intrusion Detection Using Random Forests Classifier with SMOTE and Feature Reduction. Proceedings of the 2013 International Conference on Cloud & Ubiquitous Computing & Emerging Technologies.

[15] Shon T., Moon J. (2007). A hybrid machine learning approach to network anomaly detection. Inf. Sci..

[16] Modi C., Patel D., Borisaniya B., Patel H., Patel A., Rajarajan M. (2013). A survey of intrusion detection techniques in the cloud. J. Netw. Comput. Appl..

[17] Narudin F.A., Feizollah A., Anuar N.B., Gani A. (2016). Evaluation of machine learning classifiers for mobile malware detection. Soft Comput..

[18] Hamid Y., Sugumaran M., Balasaraswathi V.R. (2016). IDS Using Machine Learning-Current State of Art and Future Directions. Br. J. Appl. Sci. Technol..

[19] Srivastava D.K., Bhambhu L. (2010). Data classification using support vector machine. J. Theor. Appl. Inf. Technol..

[20] Nasir M.U., Khan M.A., Zubair M., Ghazal T.M., Said R.A., Al Hamadi H. (2022). Single and Mitochondrial Gene Inheritance Disorder Prediction Using Machine Learning. Comput. Mater. Contin..

[21] Subbulakshmi T., Afroze A.F. Multiple learning-based classifiers using layered approach and Feature Selection for attack detection. Proceedings of the 2013 IEEE International Conference ON Emerging Trends in Computing, Communication and Nanotechnology (ICECCN).

[22] Gogoi P., Bhattacharyya D.K., Borah B., Kalita J.K. (2014). Mlh-ids: A multi-level hybrid intrusion detection method. Comput. J..

[23] Shiravi A., Shiravi H., Tavallaee M., Ghorbani A. (2012). Toward developing a systematic approach to generate benchmark datasets for intrusion detection. Comput. Secur..

[24] Buczak A.L., Guven E. (2015). A survey of data mining and machine learning methods for cyber security intrusion detection. IEEE Commun. Surv. Tutor..

[25] Hodo E., Bellekens X., Hamilton A., Tachtatzis C., Atkinson R. (2017). Shallow and deep networks intrusion detection system: A taxonomy andsurvey. arXiv.

[26] da Costa K.A., Papa J.P., Lisboa C.O., Munoz R., de Albuquerque V.H.C. (2019). Internet of things: A survey on machine learning-basedintrusion detection approaches. Comput. Netw..

[27] Ucci D., Aniello L., Baldoni R. (2019). Survey of machine learningtechniques for malware analysis. Comput. Secur..

[28] Tahsien S.M., Karimipour H., Spachos P. (2020). Machine learning basedsolutions for security of internet of things (iot): A survey. J. Ofnetwork Comput. Appl..

[29] Hussain F., Hussain R., Hassan S.A., Hossain E. (2020). Machine learningin iot security: Current solutions and future challenges. IEEE Commun. Surv. Tutor..

[30] Gibert D., Mateu C., Planes J. (2020). The rise of machine learning fordetection and classification of malware: Research developments, trendsand challenges. J. Netw. Comput. Appl..

[31] Nassif A.B., Talib M.A., Nassir Q., Nassif A.B., Talib M.A., Nasir Q., Albadani H., Dakalbab F.M. (2021). Machine learning for cloud security: A systematic review. IEEE Access.

[32] Jin Q., Wang L. Intranet user-level security traffic management withdeep reinforcement learning. Proceedings of the 2019 International Joint Conference on Neural Networks (IJCNN).

[33] Hamamoto A.H., Carvalho L.F., Sampaio L.D.H., Abrão T., Proença M.L. (2018). Network anomaly detection system using genetic algorithmand fuzzy logic. Expert Syst. Appl..

[34] Gu Y., Li K., Guo Z., Wang Y. (2019). Semi-supervised k-means ddosdetection method using hybrid feature selection algorithm. IEEE Access.

[35] Alauthman M., Aslam N., Al-Kasassbeh M. (2020). An efficient reinforcement learning-based botnet detection approach. J. Netw. Comput. Appl..

[36] Smadi S., Aslam N., Zhang L. (2018). Detection of online phishing email using dynamic evolving neural network based on reinforcement learning. Decis. Support. Syst..

[37] Xu Y., Chen N., Zhang H., Liang B. (2018). Adaptive anomaly detection strategy based on reinforcement learning. Proceedings of the International Conference of Pioneering Computer Scientists, Engineers and Educators.

[38] Sethi K., Rupesh E.S., Kumar R., Bera P., Madhav Y.V. (2020). A context-aware robust intrusion detection system: A reinforcement learning-based approach. Int. J. Inf. Secur..

[39] Rashid M.M., Kamruzzaman J., Hassan M.M., Imam T., Gordon S. (2020). Cyberattacks Detection in IoT-Based Smart City Applications Using Machine Learning Techniques. Int. J. Environ. Res. Public Health.

[40] Ofori A.Y., Swart C., Boateng F.A.O., Islam S. (2022). Cyber resilience in supply chain system security using machine learning for threat prediction. Contin. Resil. Rev..

[41] Rahman A.-U., Abbas S., Gollapalli M., Ahmed R., Aftab S., Ahmad M., Khan M.A., Mosavi A. (2022). Rainfall Prediction System Using Machine Learning Fusion for Smart Cities. Sensors.

[42] Saleem M., Abbas S., Ghazal T.M., Khan M.A., Sahawneh N., Ahmad M. (2022). Smart cities: Fusion-based intelligent traffic congestion control system for vehicular networks using machine learning techniques. Egypt. Inform. J..

[43] Nadeem M.W., Goh H.G., Khan M.A., Hussain M., Mushtaq M.F., Ponnusamy V.A. (2021). Fusion-Based Machine Learning Architecture for Heart Disease Prediction. Comput. Mater. Contin..

[44] Siddiqui S.Y., Athar A., Khan M.A., Abbas S., Saeed Y., Hussain M. (2020). Modelling, Simulation and Optimization of Diagnosis Cardiovascular Disease Using Computational Intelligence Approaches. J. Med. Imaging Health Inform..

[45] Ahmed U. (2022). Prediction of Diabetes Empowered with Fused Machine Learning. IEEE Access.

[46] Rahman A.-U., Alqahtani A., Aldhafferi N., Nasir M.U., Khan M.F., Khan M.A., Mosavi A. (2022). Histopathologic Oral Cancer Prediction Using Oral Squamous Cell Carcinoma Biopsy Empowered with Transfer Learning. Sensors.

[47] Taleb N., Mehmood S., Zubair M., Naseer I., Mago B., Nasir M.U. Ovary Cancer Diagnosing Empowered with Machine Learning. Proceedings of the 2022 International Conference on Business Analytics for Technology and Security (ICBATS).

[48] Nasir M.U., Ghazal T.M., Khan M.A., Zubair M., Rahman A.-U., Ahmed R., Al Hamadi H., Yeun C.Y. (2022). Breast Cancer Prediction Empowered with Fine-Tuning. Comput. Intell. Neurosci..

[49] Ghazal T.M., Al Hamadi H., Nasir M.U., Rahman A.U., Gollapalli M., Zubair M., Khan M.A., Yeun C.Y. (2022). Supervised Machine Learning Empowered Multifactorial Genetic Inheritance Disorder Prediction. Comput. Intell. Neurosci..

[50] Nasir M.U., Khan S., Mehmood S., Khan M.A., Rahman A.-u., Hwang S.O. (2022). IoMT-Based Osteosarcoma Cancer Detection in Histopathology Images Using Transfer Learning Empowered with Blockchain, Fog Computing, and Edge Computing. Sensors.

[51] Ray S., Sharma N., Chakrabarti A., Balas V., Martinovic J. (2021). An Analysis of Computational Complexity and Accuracy of Two Supervised Machine Learning Algorithms—K-Nearest Neighbor and Support Vector Machine. Data Management, Analytics and Innovation. Advances in Intelligent Systems and Computing.

[52] Abdiansah A., Wardoyo R. (2015). Time Complexity Analysis of Support Vector Machines (SVM) in LibSVM. Int. J. Comput. Appl..

[53] UNR-IDD Intrusion Detection Dataset. https://www.kaggle.com/datasets/tapadhirdas/unridd-intrusion-detection-dataset?resource=download.

